# Prevalence of obsessive-compulsive symptoms in patients with schizophrenia treated with clozapine: a scoping review

**DOI:** 10.1186/s12888-024-06466-9

**Published:** 2025-01-23

**Authors:** Evelyn Moreno Tarazona, Mauricio Orozco Gonzalez, Andrea La Rosa Giron, Paulo Ruiz-Grosso, Maria Lazo-Porras

**Affiliations:** 1 Facultad de Medicina, Universidad Peruana Cayetano Heredia, Lima, Peru; 2 CRONICAS Center of Excellence in Chronic Diseases, Universidad Peruana Cayetano Heredia, Lima, Peru

**Keywords:** Obsessive-compulsive disorder, Schizophrenia, Clozapine, Prevalence

## Abstract

**Background:**

Schizophrenia is a complex psychiatric disorder, and in patients treated with clozapine, it may induce or exacerbate obsessive-compulsive symptoms (OCS), which negatively affect patients’ quality of life, functionality and treatment adherence. Despite its clinical relevance, the reported prevalence and characteristics of clozapine associated OCS vary widely, limiting effective management.

**Objective:**

This scoping review synthesizes evidence on the prevalence of OCS in patients with schizophrenia treated with clozapine and explores treatment characteristics (types, severity, dose, and time to onset/exacerbation).

**Methods:**

The PRISMA-ScR methodology guided the search in PubMed, LILACS, Embase, and Scielo. Observational studies in Spanish, English, Portuguese, and French reporting prevalence, incidence, or frequency of OCS in patients over 18 years with schizophrenia treated with clozapine were included. Clinical, qualitative studies, and those with access restrictions were excluded. Risk of bias was assessed using JBI tools.

**Results:**

Fourteen studies were included, reporting OCS prevalence between 20% and 76%, and de novo OCS between 4.8% and 46.4%. Clozapine dose ranged from 196 to 525 mg/day, and treatment duration from 5 to 210 months. The most common obsessions were aggression and checking, with severity ranging from mild to moderate.

**Conclusions:**

The prevalence of OCS in patients treated with clozapine varies widely. Further research is needed to clarify the relationship between dose, treatment duration, and the onset/exacerbation of OCS.

**Supplementary Information:**

The online version contains supplementary material available at 10.1186/s12888-024-06466-9.

## Background

Schizophrenia is a complex psychiatric disorder characterized by disturbances in perception, behavior, thinking, and affect, with each individual presenting a unique symptom profile [1]. Generally, symptoms are grouped into positive symptoms, such as hallucinations, delusions, disorganized behavior, and disorganized speech; and negative symptoms, such as affective flattening, social withdrawal, and abulia [2]. Moreover, patients with schizophrenia often exhibit memory, executive functions, and mental processing speed impairment, resulting in cognitive deterioration that can occur earlier than expected for their age [3].

The treatment for this condition focuses on two main pillars: pharmacological treatment, primarily involving antipsychotic medications, and non-pharmacological treatment, consisting of psychosocial interventions aimed at restoring social and occupational functioning [4]. The choice of antipsychotic, whether typical (first-generation) or atypical (second-generation), depends on the patient’s individual characteristics and clinical presentation, as well as the properties of the medication and its side effects [5], considering that current evidence shows little difference in efficacy between first- or second-generation antipsychotics [6]. Early diagnosis and continuous treatment are essential for symptom reduction, functional maintenance, and relapse prevention [7]. Treatment-resistant schizophrenia (TRS) is defined as the persistence of positive symptoms despite treatment with at least two antipsychotics, each taken for at least six weeks with a dosage equivalent to at least 600 mg of chlorpromazine per day [8]. This condition is not uncommon, occurring in 20–25% of patients, even during the first episode of schizophrenia [9, 10].

On the other hand, obsessive-compulsive disorder (OCD) is a typically chronic and persistent condition characterized by the presence of obsessive-compulsive symptoms (OCS), which include intrusive and disturbing thoughts (obsessions) and repetitive behaviors (compulsions) that the individual feels compelled to perform [11]. It is important to note that it is possible to develop OCS without meeting the clinical criteria for OCD, as the diagnosis requires a clinical assessment by a psychiatrist according to DSM-5 criteria. Those affected by this disorder may experience a significant impact on daily life, as symptoms can be exhausting and cause considerable distress, often interfering with daily activities [12].

Obsessions can take the form of recurrent and persistent thoughts, images, or impulses that the patient perceives as invasive and absurd, often attempting to resist them but without success, causing significant discomfort. Compulsions are actions performed in response to an obsession, aimed at reducing the anxiety or discomfort associated with it. These actions may be mental, such as counting, praying, or repeating words or phrases, or physical, such as hand washing or checking something repeatedly. Compulsions are often perceived as more strange by the individual than the obsessions and are met with less resistance and greater feelings of absurdity [13].

Common types of obsessions include contamination concerns (fear of dirt, germs, diseases, and disgust toward bodily waste), safety or harm concerns (fear of being responsible for a fire), unwanted aggressive impulses (impulses to harm a loved one), unacceptable thoughts of a sexual or religious nature, and the need for symmetry or accuracy. Common compulsions include excessive cleaning (such as ritualized hand washing), repeated checking, ordering rituals, counting, repeating routine activities (such as entering and exiting a room), and hoarding (difficulty discarding objects, regardless of their actual monetary or sentimental value) [11].

Obsessions and compulsions are typically related, though approximately 20% of patients may experience pure obsessions without compulsions [13], meaning the diagnosis of OCD can be made in the absence of either [11]. It is estimated that about 2–3% of the general population suffers from OCD, with this prevalence being consistent across different cultures and geographic regions. The average age of onset for OCD is 19.5 years, though it tends to begin earlier in men than in women [13].

The onset and/or exacerbation of OCS in patients with schizophrenia is a subject of significant scientific debate, occurring at different stages of the disease. The onset of OCS has been described at various points in the course of psychotic illness: [1] before psychosis as independent or coexisting symptoms or as an OCD diagnosis [2], before the onset of psychotic symptoms as part of the at-risk mental state, i.e., as part of the prodromal symptoms of schizophrenia with a prevalence of 12.1% [3], simultaneously with the first psychotic episode with an average prevalence of 17.1% [4], after the first psychotic episode during the course of schizophrenia with an average prevalence of 25%, and [5] de novo onset after initiation of antipsychotic treatment [14]. In the case of patients with treatment-resistant schizophrenia being treated with clozapine, this medication is known to induce or exacerbate OCS in a higher proportion (38.9%) than those treated with other second-generation antipsychotics such as olanzapine (20.1%) or risperidone (23.2%) [15]. On the other hand, improvement in OCS has been observed when clozapine is reduced [16]. It should be noted that clozapine is the primary antipsychotic recommended by the American Psychiatric Association for the treatment of TRS [17]. It is also recommended for those who show a response to treatment (a reduction of at least 20% in symptoms) but still suffer a significant impact on their functionality [18], not necessarily meeting the definition of TRS.

Historically, clozapine was withdrawn from the market in the 1970s due to safety concerns related to agranulocytosis and severe neutropenia, and it was later approved for the treatment of TRS in 1990 [19]. This adverse effect is idiosyncratic, occurring in approximately 1% of patients during the first 18 weeks of treatment, most commonly within the first month [20]. Therefore, during therapy initiation, titration to a therapeutic dose is recommended to avoid this and other known adverse effects such as seizures, acute myocarditis, orthostatic hypotension, and excessive sedation. The combination of a narrow therapeutic window and significant variability in drug metabolism between patients makes titration a key factor in the occurrence of these early adverse effects [21].

The pathophysiology of OCS associated with atypical antipsychotics such as clozapine is not fully understood; however, several possible mechanisms have been proposed. First, its antiserotonergic effects due to antagonism with serotonin receptors 5-HT2A and 5-HT2C [22] could explain the fact that antipsychotic-induced OCS are associated with those with potent antiserotonergic activity, such as clozapine and olanzapine [23]. Another possible mechanism is the antidopaminergic effects on the mesolimbic system, where the neuronal circuits associated with OCD are located [24]. Furthermore, it is known that olanzapine increases glutamate release, influencing glutamatergic neurotransmission, which is related to OCS [25]. Additionally, studies suggest that the presence of polymorphisms in the SLC1A1/EAAC1 gene has been shown to be a risk factor for the development of OCS in patients with schizophrenia [14].

A review by Grillault et al. highlights the paradox concerning clozapine and other atypical antipsychotics, as they are sometimes used to treat OCD; however, in patients with schizophrenia, they may induce OCS. Therefore, the review suggests further studies to better understand the pathophysiology by observing OCS throughout the day to determine whether they are due to receptor saturation effects or dose-end effects [26].

A systematic review by Fonseka et al. in 2014 determined that the de novo onset of OCS occurred in approximately 20–28% of patients with schizophrenia treated with clozapine, and the exacerbation of OCS occurred in 10–18% [27]. This range of prevalence is influenced by various factors, such as methodological heterogeneity among studies, the type of diagnostic instrument used [28], or treatment with more than one antipsychotic, as previously reported with other antipsychotics such as aripiprazole, which is also associated with OCS and is commonly prescribed in combination with clozapine [29, 30]. A study conducted in Peru by Gálvez-Buccollini et al. [31] found that patients with schizophrenia receiving clozapine treatment exhibited significantly more OCS, particularly aggression, contamination, and miscellaneous obsessions, compared to patients treated with other antipsychotics. There was also a trend toward a higher prevalence of OCD in patients treated with clozapine compared to those treated with classical antipsychotics. Moreover, this study compared the characteristics of patients with and without OCS within the clozapine-treated group, finding that those with OCS had been on clozapine for a significantly longer period compared to those without these symptoms.

It is also important to highlight that patients with TRS are often treated with adjunctive medications that influence OCS or OCD, such as selective serotonin reuptake inhibitors (SSRIs), serotonin and norepinephrine reuptake inhibitors, or tricyclic antidepressants [27]. A systematic review of 107 case reports on clozapine-associated OCS, which evaluated 75 patients with de novo OCS and 32 with clozapine-exacerbated OCS, suggests strategies such as adding SSRIs, clomipramine, or aripiprazole to the treatment, alongside reducing clozapine dosage to improve OCS in patients. Factors associated with a good response to antidepressants for clozapine-induced OCS, such as younger age, short disease duration, short duration of clozapine treatment, and greater insight into OCS, were identified. On the other hand, factors associated with greater severity were pre-existing OCS before clozapine use and concomitant active psychosis with the presence of OCS. While this review provided important insights, it is not possible to estimate the prevalence of clozapine-associated OCS based on the reported results, as they were derived from case reports and case series [32]. OCS associated with schizophrenia, as an additional comorbidity, have a significant impact on patients’ social functioning and quality of life, considerably reducing patients’ well-being, as well as interfering significantly with individual functionality and treatment adherence, negatively affecting prognosis and therapeutic outcomes [33].

Accurately determining the proportion of patients receiving clozapine who develop OCS or obsessive-compulsive disorder OCD is critical for estimating the prevalence of these symptoms and understanding the treatment characteristics that may influence their development, such as the average time to onset or exacerbation and the typical dosages used. However, despite the clinical relevance of this phenomenon, there is significant variability in the reported prevalence and characteristics of clozapine-associated OCS, complicating data interpretation and hindering effective management strategies. Furthermore, no recent systematic reviews have specifically evaluated the impact of second-generation antipsychotics on OCS in patients with schizophrenia or consistently reported the prevalence of these symptoms. Therefore, a comprehensive scoping review is urgently needed to synthesize current evidence and provide an estimate of clinically significant OCS prevalence in this population. The findings will be instrumental in optimizing management strategies, ultimately improving the quality of life and therapeutic outcomes for these patients by offering critical evidence to inform clinical decisions.

## Objectives

### Main objective

To estimate the prevalence of clinically significant obsessive-compulsive symptoms (OCS) in patients with schizophrenia undergoing clozapine treatment.

### Secondary objectives

- To estimate the prevalence of de novo OCS/OCD in patients undergoing clozapine treatment.
- To estimate the prevalence of OCS exacerbation in patients undergoing clozapine treatment.
- To describe the characteristics of clozapine treatment in patients with OCS (average treatment duration until symptom onset or exacerbation, and average treatment dosage).
- To describe the characteristics of OCS (type and severity).

## Methods

### Study Design

This study follows a scoping review design, a type of evidence synthesis. The methodology was guided by the Preferred Reporting Items for Systematic Reviews and Meta-Analyses extension for Scoping Reviews (PRISMA-ScR) [34], as outlined in Appendix 1.

### Protocol registration

A protocol for this scoping review was developed, which is not available in a public database; however, it has been reviewed and registered with the University Directorate for Regulatory Affairs of Research at the Universidad Peruana Cayetano Heredia.

### Eligibility criteria

#### Inclusion criteria

##### Study types

Descriptive or analytical observational studies, either cross-sectional or longitudinal, that report obsessive-compulsive symptoms (OCS) and have a representative sample of the study population, published in Spanish, English, Portuguese, or French.

##### Participants

Studies were included if they involved patients aged 18 years or older with a diagnosis of any type of schizophrenia (ICD-10: F20.0 to F20.9, DSM-5 295.90, or corresponding DSM classifications relevant at the time of study publication) undergoing treatment with clozapine in any form, at any dose, and for any duration, whether in outpatient or inpatient settings.

##### Outcome measurement

Studies with any method of OCS detection were included, whether using obsessive-compulsive symptom scales or through clinical diagnosis.

##### Time Period

No time restrictions were applied to the literature search for this scoping review, as all available literature was considered.

#### Exclusion criteria

Clinical trials, case-control studies, case series, case reports, letters to the editor, editorials, and qualitative studies were excluded. Additionally, studies that could not be accessed due to restricted availability were excluded.

### Information sources

The literature search was conducted on August 22, 2024, using the bibliographic databases PubMed, LILACS, Scielo, and Embase. Systematic and narrative reviews were also explored to perform a complementary search through reference lists.

### Search strategy

For the search strategy in this investigation, a variant of the PICO format known as CoCoPo (Condition, Context, Population) was used to formulate the research question, as detailed in Table 1.

**Table 1:** CoCoPo Format

CoCoPo Format	
**Condition**	Obsessive-compulsive symptoms
**Context**	Prevalence
**Population**	Patients with schizophrenia treated with clozapine
**Research question**	What is the prevalence of obsessive-compulsive symptoms in patients with schizophrenia treated with clozapine?

The search for relevant studies was conducted in the bibliographic databases described in the previous section. In PubMed, search terms were based on Medical Subject Headings (MeSH) and related free terms; in LILACS and Scielo, Health Sciences Descriptors (DeCS) and related free terms were used. The terms were combined using Boolean operators OR and AND. Finally, in Embase, Emtree terms were employed along with related free terms. The full search strategy is detailed in Appendix 2.

### Selection of evidence sources

The reference management software Rayyan QCR [35] was used to import, store, and select the bibliographic search results from each database. Separate folders were created for each database to maintain the original search results. Then, all folders were merged into one to proceed with the removal of duplicate articles. Three reviewers, working in pairs, screened titles and abstracts. The full-text screening was also performed in pairs. Disagreements in study selection were resolved through discussion and consensus with a third reviewer.

### Data collection process

The data extraction process for each study was carried out independently and in duplicate by two researchers. The extracted data were then compared, and any discrepancies were resolved by consensus, involving a third researcher when necessary. The data collected included both general study information and the outcomes of interest, which are detailed below.

### Review data

The information collected from each study was categorized into two main sections:

#### General Information

- **Author**: Name of the study’s primary author.
- **Title**: Original title of the study in the language of publication.
- **Design**: Methodological design of the study.
- **Population**: Description of the group of interest from which the sample was drawn.
- **Objective**: Main objective of the study.
- **Country**: Country where the study was conducted.
- **Language**: Language in which the study was published.

#### Outcomes of interest

- **Sample size**: Number of patients included in the study.
- **Average age**: Average age of the participants, expressed in years.
- **Male sex (%)**: Percentage of males in the study sample.
- **Average dose of clozapine (mg/day)**: Mean daily dose of clozapine administered to the patients in the sample.
- **Treatment duration (months)**: Duration of clozapine treatment in months.
- **Prevalence of SOC/TOC**: Number of patients with obsessive-compulsive symptoms (SOC) or obsessive-compulsive disorder (OCD) in the sample.
- **Prevalence of de novo SOC/TOC**: Number of cases of OCS/OCD diagnosed after starting treatment with clozapine.
- **Prevalence of exacerbation of previous SOC/TOC**: Number of patients with pre-existing OCS/OCD whose symptoms worsened during treatment.
- **Scale used**: Tool used to assess the presence and severity of SOC/OCD, as well as the score obtained, where applicable.

#### Scales for evaluating OCS/OCD

- **Yale-Brown Obsessive-Compulsive Scale (Y-BOCS)**: A widely used scale for assessing the severity of OCS, consisting of 10 items (five related to obsessions and five to compulsions). Each item is scored from 0 to 4, with a total score ranging from 0 to 40. Severity is categorized into four levels: mild [8–15], moderate [16–23], severe [24–31], and extreme [32–40]. Additional versions exist, such as the Y-BOCS-II, which aims to improve the accuracy of evaluations [37].
- **Maudsley Obsessive-Compulsive Inventory (MOCI)**: A questionnaire that assesses the presence and severity of OCS through 30 dichotomous items (present/not present), distributed across four subscales: checking, cleaning, slowness, and doubt [38].
- **Obsessive-Compulsive Inventory (OCI)**: Measures OCS severity with 42 items divided into seven subscales (washing, checking, doubt, ordering, obsession, hoarding, and neutralizing), each scored from 0 to 4 [39].
- **Obsessive-Compulsive Inventory - Revised (OCI-R)**: An abbreviated version of the OCI that assesses OCS severity through 18 items, organized into six subscales [40].
- **National Mental Health Institute Global Obsessive-Compulsive Scale (NIMH-GOCS)**: A global scale that measures overall OCS severity using a single item, with a score ranging from 1 to 15 [41, 42].

### Critical assessment of methodological quality

The quality of the studies included in this scoping review was assessed using the JBI critical appraisal tools [43]. This tool is used to determine the validity, relevance, and potential biases of the studies included in systematic reviews and other types of evidence synthesis, such as this study.

Each JBI tool consists of a series of questions according to the type of study. In our case, since the included studies were cross-sectional, we used the JBI’s Critical Appraisal Checklist for Analytical Cross-Sectional Studies.

### Synthesis of results

To synthesize the results of our scoping review, we grouped the included studies according to the research objectives and the characteristics of the reported data. We summarized the results as follows: prevalence of OCS prevalence of de novo OCS, prevalence of exacerbation of OCS, characteristics of clozapine treatment (dose and duration), and characteristics of OCS.

## Results

### Search results

A search was conducted in the mentioned databases, yielding 613 results in PubMed, 160 results in LILACS, 699 results in Embase, and 0 results in Scielo, for a total of 1,472 results. These results were imported into the reference management program Rayyan, where the program detected 567 possible duplicates. Subsequently, 327 articles that were indeed duplicates were removed through manual review.

**Fig. 1:**
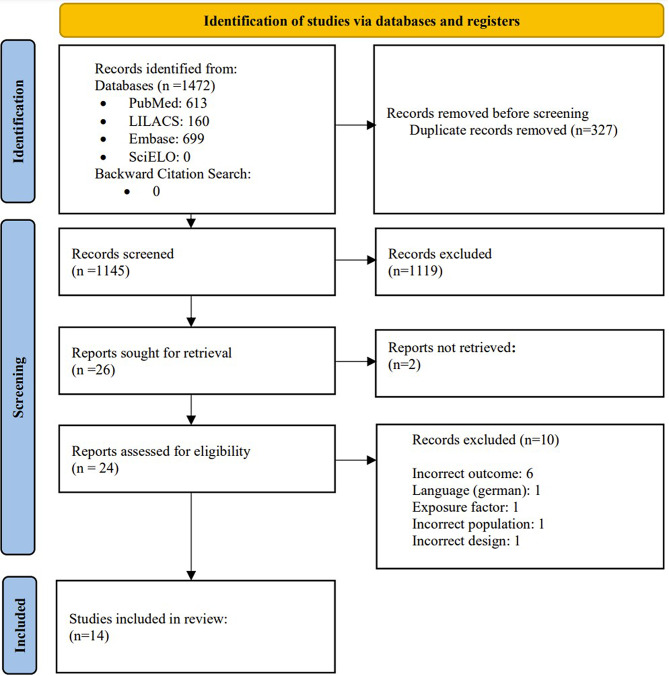
PRISMA flowchart

The remaining 1,145 articles were evaluated independently by two reviewers for selection based on title and abstract. As a result of this evaluation, 26 articles were selected for full review. During the full-text review, the authors of two selected articles from the title and abstract screening phase were contacted; however, both articles could not be obtained [44, 45], and ten were excluded for not meeting the inclusion and exclusion criteria, leaving a final count of 14 articles. It is noteworthy that no results were obtained in the complementary search by references (Fig. 1).

### General characteristics of the studies

A total of 14 studies were included in this review, of which 13 were published in English and 1 in Spanish, with the oldest published in 1995 and the most recent in 2024 [28, 31, 46–57]. All studies included in the review had a cross-sectional design and were conducted in the countries of Germany (*n* = 1), Brazil (*n* = 1), China (*n* = 1), the United States (*n* = 1), Italy (*n* = 1), Ireland (*n* = 1), Peru (*n* = 1), the United Kingdom (*n* = 4), Singapore (*n* = 1), and Turkey (*n* = 2). The primary objective of half of the studies was to determine the prevalence of OCS in patients diagnosed with schizophrenia receiving clozapine treatment, while the remaining half reported it as part of their results (Table 2).

**Table 2:** General characteristics of the studies

Autor	Título	Diseño	Población	Objetivo	País	Idioma
**Ghaemi et al.** **1995**	Is there a relationship between clozapine and obsessive-compulsive disorder?: A retrospective chart review	Descriptive retrospective analysis	Clinical records from patients who were admitted to McLean Hospital before July, 1992, and started on clozapine treatment.	To evaluate the incidence of emergence of obsessive-compulsive symptoms during treatment with clozapine.	USA	English
**Gálvez-Buccollini et al.** **2004**	Síntomas obsesivo- compulsivos en esquizofrenia durante el tratamiento con clozapina y antipsicóticos clásicos	Cross-sectional study	Patients diagnosed with schizophrenia undergoing clozapine treatment or atypical antipsychotic, attendet at the outpatients clinic of National Institute of Mental Health from December 2002 to January 2003.	To compare the prevalence of obsessive-compulsive symptoms in schizophrenic patients in treatment with clozapine and those who receive classic antipsychotic drugs.	Peru	Spanish
**Ertugrul et al.** **2005**	Obsessive-compulsive symptoms in clozapine-treated schizophrenic patients	Descriptive cross-sectional study	Patients with schizophrenia who were receiving clozapine treatment at least for the past 6 weeks	To assess the occurrence of obsessive-compulsive symptoms (OCS) in schizophrenic patients treated with clozapine, and to examine the relationship between OCS and other clinical variables.	Türkiye	English
**Lin et al.** **2006**	Higher plasma drug concentration in clozapine-treated schizophrenic patients with side effects of obsessive/compulsive symptoms	Retrospective and naturalistic study	Schizophrenic patients treated with clozapine during a 1-year study period.	To investigate the incidence of these side effects, together with the relation between these side effects and the plasma concentration of clozapine and its metabolites norclozapine and clozapine-N-oxide in schizophrenic patients.	China	English
**Sa et al.** **2009**	Obsessive-compulsive symptoms and disorder in patients with schizophrenia treated with clozapine or haloperidol	Cross-sectional study	Patients who met diagnostic criteria for schizophrenia according to the DSM-IV. Patients in use of clozapine were recruited from the outpatient clinic in São Paulo, Brazil, between July and December 2006. Patients taking haloperidol were recruited from the schizophrenia outpatient clinic at the same hospital.	To compare the prevalence and severity of obsessive-compulsive symptoms (OCSs) and obsessive-compulsive disorder (OCD) in patients with schizophrenia treated with clozapine or haloperidol.	Brazil	English
**Bleakley et al.** **2011**	Does clozapine cause or worsen obsessive Compulsive symptoms? An analysis and literature review	Descriptive cross-sectional study	Patients currently registered for clozapine treatment for at least one year in the Southampton Area, UK.	To further investigate the complex relationship between clozapine and OCS.	UK	English
**Grassi et al.** **2013**	Hypochondriasis and obsessive-compulsive disorder in schizophrenic patients treated with clozapine vs. other atypical antipsychotics	Descriptive cross-sectional study	Schizophrenic patients treated with clozapine or other atypical antipsychotics (olanzapine, quetiapine, risperidone, aripiprazole, amisulpride, and paliperidone) who met diagnostic criteria for schizophrenia according to the DSM-IV	To investigate the prevalence rates of obsessive-compulsive disorder (OCD) and hypochondriasis in schizophrenic patients treated with atypical antipsychotics (AAPs) and to investigate the different comorbidity rates of OCD and hypochondriasis between clozapine-treated patients and patients treated with other AAPs.	Italy	English
**Doyle et al.** **2014**	Obsessive compulsive symptoms in patients with Schizophrenia on Clozapine and with obsessive compulsive disorder: A comparison study	Cross sectional observational study	A list of patients registered with the clozapine monitoring service was compiled by cross checking registered patients with the pharmacy service, a nurse led monitoring clinic and consultant lists of patients. The comparison group was recruited from patients referred to a cognitive behavior therapy group for obsessive compulsive disorder.	This study describes the obsessive-compulsive symptom profile of a population of patients with schizophrenia treated with clozapine and compares this with patients with obsessive compulsive disorder.	Ireland	English
**Fernandez-Egea et al.** **2018**	Distinct risk factors for obsessive and compulsive symptoms in chronic schizophrenia	Cross-sectional study	The electronic records of a large cohort of clozapine-medicated schizophrenia patients at the Cambridgeshire and Peterborough NHS Foundation	To address three questions of OCD prevalence, clinical profile and associated severity factors, using electronic records of a large cohort of clozapine-medicated schizophrenic patients.	UK	English
**Gürcan et al.** **2021**	Clinical risk factors, phenomenology and the impact of clozapine induced obsessive compulsive symptoms	Cross-sectional and retrospective study	Patients of the Department of Psychiatry, Hacettepe University School of Medicine receiving clozapine treatment for at least 6 weeks	To investigate the clinical risk factors, phenomenology and the impact of clozapine induced obsessive-compulsive symptoms (OCS) in patients with schizophrenia.	Türkiye	English
**Parkin et al.** **2022**	Clozapine-related obsessive-compulsive symptoms and their impact on wellbeing: a naturalistic longitudinal study	Cross-sectional and retrospective study	Anonymized electronic healthcare records from a large cohort of patients who were treated with clozapine and assessed annually for OCS, wellbeing, general functioning, and psychopathology using standardized scales as part of routine clinical practice.	To evaluate the longitudinal influence of OCS severity on wellbeing and general functioning.	UK	English
**Seow et al.** **2023**	Obsessive-compulsive symptoms and disorder in clozapine-treated schizophrenia	Cross-sectional study	Patients from the Institute of Mental Health (IMH) in Singapore, with a diagnosis of schizophrenia who are currently prescribed clozapine and have had no changes to current prescription for the past 2 weeks.	To report the prevalence of OCS and OCD, and examine potential risk factors, in clozapine-treated schizophrenia.	Singapore	English
**Fernandez-Egea et al.** **2024**	The role of psychosis and clozapine load in excessive checking in treatment-resistant schizophrenia: longitudinal observational study.	Naturalistic longitudinal observational study	Anonymised electronic records gathered by Cambridgeshire and Peterborough NHS Foundation Trust (CPFT).	To use habit formation models developed in cognitive neuro- science to investigate the dynamic interplay between psychosis, clozapine dose and obsessive–compulsive symptoms (OCS).	UK	English
**Morgenroth et al.** **2024**	Polygenetic risk scores and phenotypic constellations of obsessive-compulsive disorder in clozapine-treated schizophrenia.	Descriptive analyses	Department of Psychiatry and Neurosciences at the Charité – Universitätsklinikum Berlin and at the Department of Psychiatry and Psychotherapy at the University Hospital of Munich (LMU Munich), in both inpatient and outpatient clinical settings from May 2017 to March 2020.	To analyze the prevalence of OCS and obsessive –compulsive disorder (OCD) in individuals with schizophrenia (SCZ) treated with clozapine and find possible correlations with different phenotypes. To examine polygenetic risk scores (PRS) in individuals with schizophrenia and OCS.	Germany	English

The data collection method in three of the 14 studies was solely through medical records [46, 50, 56], one study used only patient interviews [51], six studies utilized both medical records and interviews [28, 31, 47, 49, 52, 54], and finally, four studies used interviews, medical records, and blood sample analysis [48, 53, 55, 57], of which three aimed to determine plasma clozapine concentration and one to assess the genes of the sample [57]. It is noteworthy that two of the studies conducted interviews not only with patients but also with caregivers or family members [48, 53]. A total of ten studies evaluated medical records and/or outpatient patients [31, 47–49, 51–56], one study evaluated medical records of hospitalized patients only [46], two studies evaluated medical records of both outpatient and hospitalized patients [50, 57], and one study did not specify whether the medical records evaluated were from outpatient or hospitalized care [28].

The number of participants in the studies ranged from 49 to 196 participants, with an average age ranging from 30 to 47 years, and a higher proportion of male participants. Regarding the characteristics of clozapine treatment, the average dose ranged from 196 to 525 mg/day, and the duration of treatment ranged from five to 210 months.

The samples of the reviewed studies were diverse: seven of them included only patients diagnosed with schizophrenia receiving clozapine treatment [28, 47, 48, 53–56]; while in four other studies, in addition to patients with schizophrenia, patients with other diagnoses such as schizoaffective disorder, schizophreniform disorder, bipolar disorder, borderline personality disorder, psychotic depression, and undifferentiated psychosis were included [50, 52, 56, 57]. On the other hand, the remaining three studies included patients with a diagnosis of schizophrenia who were treated not only with clozapine but also with other atypical antipsychotics such as olanzapine, quetiapine, risperidone, etc., and classical antipsychotics such as haloperidol [31, 49, 51].

Two studies addressed family history in their results. The first, by Ertugrul et al. [47], reported no significant differences in the family history of mental illness between the groups. The second, by Mongenroth et al. [57], indicated that 57% of participants had a family history of psychiatric disorders; however, this finding was not further discussed in their analysis.

### General prevalence of OCS/OCD in patients with schizophrenia treated with clozapine

The prevalence of OCS was identified in seven of the 14 studies [47–49, 52, 54–57]. To evaluate the overall prevalence of OCS, a comparison was made between the prevalences reported in the seven studies. The observed prevalences ranged from 20 to 76%, with a mean of 36.86 ± 19.51. To provide a more robust measure of central tendency and dispersion against the variability of the data, the median was calculated at 38%, with an interquartile range (IQR) of 18%.

**Fig. 2:**
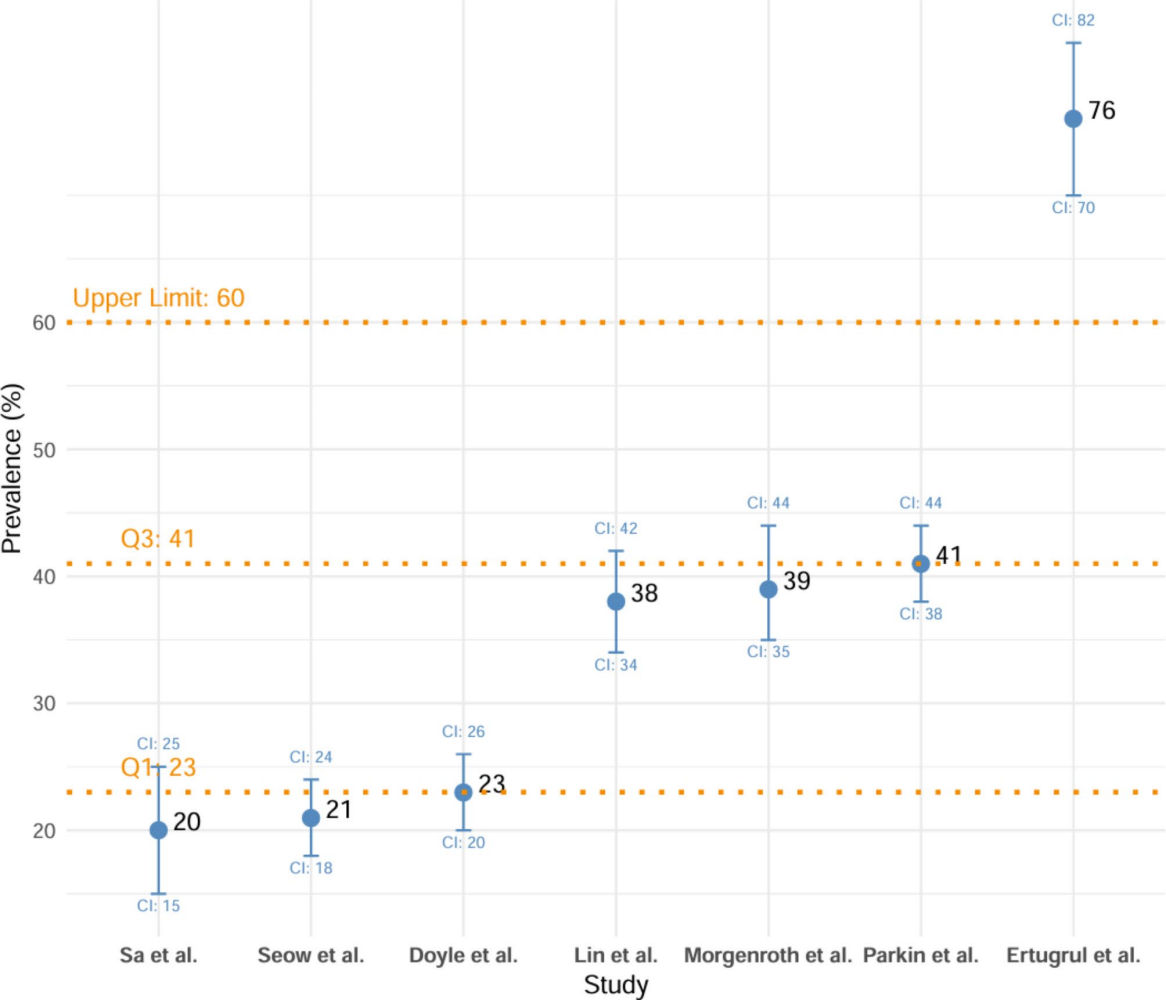
Scatter plot of general prevalence rates of OCS across studies

The scatter plot (Fig. 2) shows the prevalences from the eight studies. An outlier with a prevalence of 76% was reported by Ertugrul et al. [47], which is considerably higher than most of the other articles. Additionally, one of the eight studies used comparator groups of clozapine versus another antipsychotic in its methodology: Sa et al. [49], which, in addition to clozapine, evaluated the prevalence of OCS in patients treated with haloperidol, reporting prevalences of 20% and 25%, respectively.

**Fig. 3:**
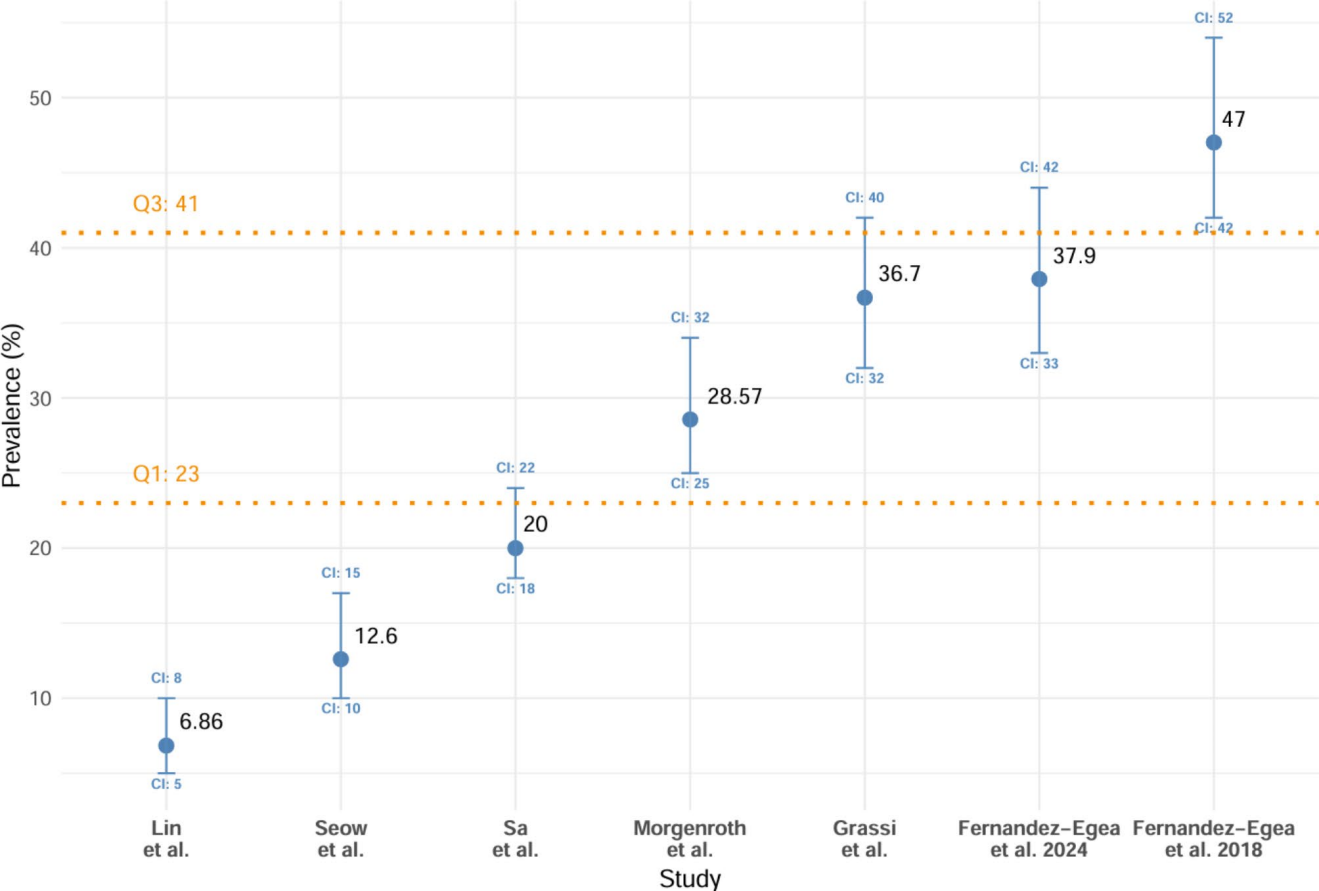
Scatter plot of general prevalence rates of ocd across studies

**Fig. 4:**
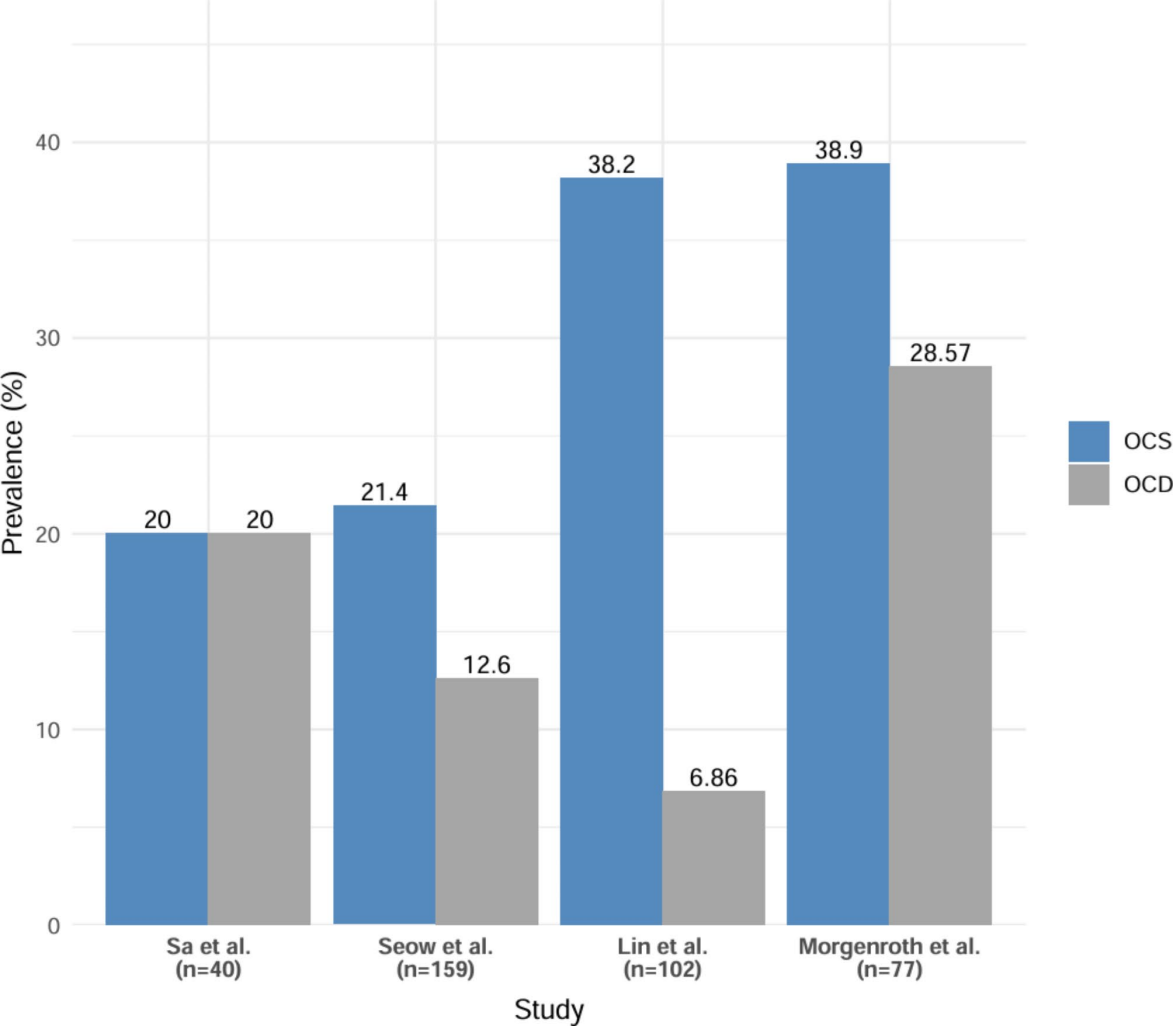
Comparison of prevalence of OCS and OCD by study

The overall prevalence of OCD was reported in seven studies [28, 48, 49, 51, 55, 57] (Fig. 3). This was found to range from 6.9 to 47%. The mean was 27 ± 14.6, the median was 28.5%, and the IQR was 21%. A comparison between overall prevalences of OCS and OCD in studies that reported these results is shown in (Fig. 4).

When evaluating the seven studies that included only patients with schizophrenia treated with clozapine [28, 47, 48, 53–56], without considering studies with comparator groups using other drugs or different clinical diagnoses, a prevalence of OCS ranging from 21.4 to 76% was observed, with a median of 41% and an IQR of 7.6. Four of these reviewed studies reported the concomitant use of medication [28, 47, 48, 53]. Fernández-Egea et al. [28] evaluated the use of medications with potential effects on OCD, including aripiprazole, SSRIs, serotonin and norepinephrine reuptake inhibitors (SNRIs), and tricyclic antidepressants (TCAs), referred to in the study as “anti-OCD medication.” The results indicated that the prevalence of OCD and the mean OCI-R scores in patients using anti-OCD medication were significantly higher compared to those taking only clozapine (64% vs. 31%; *p* = 0.001) and (24.6 ± 13.2 vs. 16.2 ± 12.2; *p* = 0.001). In the study by Ertugrul et al. [47], with a sample of 50 patients, it was reported that ten were on adjunctive treatment with SSRIs, of which three received it for comorbid OCD and another three for comorbid major depression. On the other hand, Lin et al. [48] analyzed the prevalence of medications that affect clozapine blood concentrations, including fluvoxamine, fluoxetine, sertraline, paroxetine, and valproic acid. The analysis showed that, out of 39 patients with OCS, 17 (43.6%) were taking this type of medication, while only 11 out of 63 (17.5%) without OCS were using it, evidencing a significant difference in prevalences. Furthermore, Gürcan et al. [53] reported that 62 patients in their sample were taking serotonin reuptake inhibitors (SRIs), including SSRIs, SNRIs, and TCAs. The results showed that patients receiving SRIs had a significantly higher mean score on the YBOCS scale compared to those not taking these medications (15 ± 10.56 vs. 8.25 ± 8.43; *p* < 0.001). The study also reported that 31 patients were taking another antipsychotic in addition to clozapine, 11 took benzodiazepines, and 16 took antiepileptics and/or mood stabilizers.

### Prevalence of *de novo* OCS/OCD in patients with schizophrenia treated with clozapine

In the analysis of the prevalence of de novo OCS, data from five studies were collected (Table 3). These data show notable variability in the frequency of de novo OCS among the reviewed studies. The observed prevalence range varies from 4.8 to 46.4%. The median was 20% and the IQR was 9%. Galvez-Bucollini et al. [31] evaluated the prevalence of de novo OCS in treatment with clozapine and classical antipsychotics.

**Table 3:** Summary of study results

Author	Sample size	Mean age (SD)	Male sex (%)	Mean clozapine dose in mg/day (SD)	Duration of clozapine treatment in months (SD)	Prevalence of OCS/OCD	Prevalence of de novo OCS/OCD	Prevalence of exacerbation of preexisting OCS/OCD	Scale used and score according to scale (SD)
Gürcan et al.	Total: 122 Group A: 54 developed de novo OCS Group B: 41 with preexisting OCS Group C: 26 without OCS	Total: 41.4 (10.65) Group A: 42.39 (10.17) Group B: 38.37 (10.52)	Total: 65 (53.3%) Group A: 32 (59.3%) Group B: 23 (56.1%)	Total: 381,35 (163.69) Group A: 393,06 (182) Group B: 351.22 (156.72)	Total: 110.08 (66.74) Group A: 120,04 (63.4) Group B: 103.48 (6542)	-	Group A: 54 (44.3%)	-	Y-BOCS: Group A: 13.74 (9.17) Group B: 16.66 (8.67)
**Sa et al.**	Total: 60 CLZ: 40 HAL: 20	CLZ: 35,15(8.87) HAL: 33,3(10.12)	Total: 45 (75%) CLZ: 31 (77.5%) HAL: 14 (70%)	-	-	**OCS**: CLZ: 8 (20%) HAL: 5 (25%) **OCD**: CLZ 8: (20%) HAL: 2 (10%)	-	-	Y-BOCS (OCS + OCD): CLZ: 21.5 (6.79) HAL: 12.71 (7.99)
**Grassi et al.**	Total: 60 CLZ: 30 AAP: 30	Total: - CLZ: 39.87 (9.95) AAP: 47.60 (10.30)	Total: 34 (56.7%) CLZ: 17 (56.7%) AAP: 17 (56.7%)	-	CLZ: 72.84 (44.04)	**OCS**: - **OCD**: CLZ: 11 (36.7%) AAP: 5 (16.7%)	-	-	Y-BOCS: CLZ: 10.9 (9.18) AAP: 5.9 (8.31)
**Lin et al.**	Total: 102 Without OCS: 63 OCS: 39	Total: 37,9 (9,3) Without OCS: 37,1(9,5) OCS: 39,1(9,0)	Total: 59 (57,8%)	Total: - Without OCS: 268,9(110,1) OCS: 305,1(157,2)	Total: 65,9 (39,5) Without OCS: 56,1(40,6) OCS: 81,8(32,2)	**OCS**: 39 (38,2%) **OCD**: 7 (6,86%) *	**OCS**: 29 (28%) **OCD**: 6 (5,88%)*	**OCS**: 9,8% (10) **OCD**: 1 (0,98)*	**OCS**: Y-BOCS: 10,0 (4,7) NIMH-GOCS: 6,0 (3,2)
**Gálvez-** **Bucollini et al.**	Total: 110 CLZ: 56 TAP: 54	Total: - CLZ: 30,04(6,69) TAP: 33,63(10,87)	Total: 83 (75,5%) CLZ: 47 (83,9%) TAP: 36 (66,6%)	Total: 196,01 (96,43)	Total: 34,66 (23,92)	**-**	**SOC**: CLZ: 26 (46,4%) TAP: 11 (20,4%)	-	Y-BOCS: -
**Morgenroth et al.**	Total: 91 SCZ: 77	Total: 42,77 (10,57) SCZ: -	Total: 79 (63,2%) SCZ: -	Total: 244,03 (143,74)	Total: 117,6 (106,8)*	**SOC**: SCZ: 30 (38,9%) **TOC**: SCZ: 22 (28,57%*)	-	-	Y-BOCS: -
**Seow** et al.	159	OCS: 36,4 (10,0) OCD: 36,8 (8,7)	102 (64,2%)	OCS: 337,5 (165,3) OCD: 325,0 (148,5)	OCS: 54.83 (60,8) OCD: 59,03 (54,6)	**OCS**: 34 (21,4%) **OCD**: 20 (12,6%)	-	-	Y-BOCS-II
**Doyle et al.**	SCZ + CLZ: 62 OCD: 35	SCZ + CLZ: 38,32 OCD: 38,94	SCZ + CLZ: 42 (70%) OCD: 16 (45,7%)	SCZ + CLZ: 385,6 (140,2)	-	**OCS**: SCZ + CLZ: 14 (23%)	**OCS**: SCZ + CLZ: 12 (19%)	-	OCI: SCZ + CLZ: 41,04 (30,59) OCD: 71,77(29,56)
**Fernandez-Egea et al.** **2018**	118	44,3 (11,0)	98 (83%)	346 (141)	175,2 (85,2)*	**OCD**: 55 (47%)	-	-	OCI-R: 20,3 (13,7)
**Parkin** et al.	184	45,9 (10,9)	147 (79,9%)	318,0 (141,9)	195,72 (117,6)*	**OCS**: 75* (41%)	-	-	OCI-R: 18,95 (13,4)
**Fernandez-Egea et al.** **2024**	196	47.44 (10.42)	155 (79,1%)	335,8 (145,0)	210.12 (106,92)*	**OCS**: 74 (37,9%)	-	-	OCI-R total: 18,44 (13,55)
**Ertugrul et al.**	50	35 (11,2)	28 (56%)	408 (178,8)	45 (36,1)	**OCS**: 38 (76%)	**OCS**: 10 (20%)	**OCS**: 9 (18%)	MOCI: 18,6(7,6)
**Bleakley et al.**	Total: 49 SCZ: 42	-	Total: 34 (69,4%) SCZ: -	Total: 483 SCZ: 525*	Total: 5–9 SCZ: 5–9*	-	**OCS**: Total: 6% SCZ: 4,8%*	-	-
**Ghaemi et al.**	Total: 142 SCZ: 41	Total: 34 (12,2)	Total: 80 (56,3%)	Total: 291,2 (183,9)	Total: 5,05 (7,8)	-	-	**OCD**: SCZ: (1) 2,43%	-

*Calculation performed by the researchers

SCZ: Patients with diagnosis of schizophrenia

CLZ: Clozapine

TAP: Typical antipsychotics

AAP: Atypical antipsychotics

SD: Standard deviation

HAL: Haloperidol

OCS: Obsessive-compulsive symptoms

OCD: Obsessive-compulsive disorder

n: Number of participants

Y-BOCS: Yale-Brown Obsessive-Compulsive Scale

Y-BOCS-II: Yale-Brown Obsessive-Compulsive Scale 2° edition

MOCI: Maudsley Obsessive-Compulsive Inventory

NIMH-GOCS: National Mental Health Institute Global Obsessive-Compulsive Scale

OCI: Obsessive-Compulsive Inventory

OCI-R: Obsessive-Compulsive Inventory - Revised

Only one study identified the prevalence of de novo OCD among its participants with a value of 5.9% [48]. This study analyzed the prevalence of clozapine-induced OCS/OCD and its correlation with the concentration of clozapine and its metabolites in plasma from a population of patients diagnosed with schizophrenia undergoing treatment with clozapine from outpatient and daycare clinic settings at a psychiatric center. It evaluated a sample of 102 patients, with 39 patients having OCS and 63 patients without OCS. Of the group of patients with OCS, OCD was diagnosed in seven of them.

### Prevalence of exacerbation of OCS/OCD in patients with schizophrenia treated with clozapine

Two studies were identified that reported the prevalence of exacerbation of OCS in their samples. Lin et al. [48] reported an OCS exacerbation prevalence of 10%, and Ertugrul et al. [47] reported an OCS exacerbation prevalence of 18%. The latter evaluated the occurrence of OCS in a sample of 50 patients from a population with treatment-resistant schizophrenia who had received at least six weeks of treatment with clozapine. The study conducted by Lin et al. [48] identified within their sample one patient who experienced worsening OCD symptoms, showing a prevalence of 1%.

### Assessment of obsessive-compulsive symptoms

There is considerable variability among the studies regarding how OCS are defined, which can impact the interpretation and comparison of findings across studies. As shown in Table 4, some studies use specific diagnostic criteria or standardized scales, while others rely on clinical observations or self-reported symptoms.

**Table 4:** Definition for OCS/OCD in each study

Author		Definition	
Ghaemi et al.	OCS	Presence of either obsessions or compulsions of an ego-dystonic character as defined in DSM-III-R	
	OCD	DSM-III-R	
Gálvez-Buccollini et al.	OCS	Obsessive symptoms: recurrent and persistent ideas, thoughts, images or impulses that the subject considered intrusive or inappropriate and whose content was bothersome and sometimes even shameful for oneself Compulsive symptoms: repeated behaviors performed according to certain rules or stereotypical forms to decrease anxiety, that could be behavior compulsions (observable rituals) or cognitive compulsions (non-observable rituals that occur in the mind of the subject) OCS: at least one of the two previously mentioned symptom types	
	OCD	Yale-Brown Checklist of Obsessive-Compulsive Symptoms and Structured Clinical Interview for DSM-IV	
Ertugrul et al.	No explicit definitions for OCS mentioned. Maudsley Obsessive-Compulsive Inventory was used for the assessment of symptoms		
Lin et al.	No explicit definitions for OCS mentioned, a screening questionnaire for obsession and compulsion was administered to all patients, if shown any symptom, the Yale-Brown Obsessive-Compulsive Scale and National Institute of Mental Health Global Obsessive-Compulsive Scale were administered for the assessment of symptoms		
Sa et al.	OCS	No explicit definition mentioned, the severity of the symptoms was measured using the Yale-Brown Obsessive-Compulsive Scale	
	OCD	Structured Clinical Interview for DSM-IV Axis I disorders-patient edition	
Bleakley et al.	Three markers of OCS were chosen: 1. Any record of an International Classification of Disease (ICD) or Diagnostic Statistical manual (DSM) diagnosis of OCD. 2. Any record of the follow symptoms (obsessions, obsessional thoughts, ruminations, repetitive impulses, compulsions, repetitive thoughts or actions, repetitive behavior or rituals). 3. Any prescribing of a selective serotonin reuptake inhibitor (SSRI), venlafaxine or clomipramine.		
Grassi et al.	OCS	Presence of OC symptoms by the Yale–Brown Obsessive-Compulsive Scale	
	OCD	Structured Clinical Interview for DSM-IV Axis I Disorders	
Doyle et al.	OCS	No explicit definitions for OCS mentioned, Obsessive Compulsive Inventory was used as a self-report measure of OCS	
	OCD	DSM-IV diagnosis of OCD based on clinical assessment	
Fernandez-Egea et al. 2018	OCS	No explicit definition mentioned	
	OCD	Obsessive Compulsory Inventory-Revised with the cut-off point for OCD diagnosis of 21 for the total OCI-R or a cut-off point of 5 for any of the 6 factors of the scale	
Gürcan et al.	OCS	Any symptom in Yale-Brown Obsessive Compulsive Symptom Checklist	
	OCD	Structured Clinical Interview for DSM-IV Axis I Disorders - SCID-I	
Parkin et al.	No explicit definitions for OCS mentioned. The severity of OCS was measured by the Obsessive-Compulsive Inventory-Revised		
Seow et al.	OCS	The Yale-Brown Obsessive–Compulsive Scale – second edition was used to evaluate presence and severity of OCS, where the presence of OCS was defined as the combined score of ≥ 1	
	OCD	Structured Clinical Interview for DSM-IV-TR	
Fernandez-Egea et al. 2024	OCS	No explicit definition mentioned	
	OCD	Obsessive-Compulsive Inventory - Revised was used with a total score ≥ 21 or ≥ 5 on any subscale	
Morgenroth et al.	OCS	A cutoff score of 8 in the Yale-Brown Obsessive–Compulsive Scale	
	OCD	A cutoff score of 13 in the Yale-Brown Obsessive–Compulsive Scale	

#### Findings by measurement scales

- **Y-BOCS**: A total of six studies employed the Y-BOCS as an instrument for the assessment of OCD [31, 48, 49, 51, 53, 57], making it the most frequently used scale among all studies included in the review. Of these six studies, the mean total score is specified in the results of 4 of them [48, 49, 51, 53], which ranges from 10 ± 4.7 to 21.5 ± 6.7.
- **Y-BOCS-II**: One study used the Y-BOCS-II scale [55] in its analysis, establishing a cutoff point of ≥ 1 for the presence of OCD; it found that 21.4% of its sample presented OCD, although the mean total score is not detailed in its results.
- **MOCI**: One study employed this scale as an instrument for measuring OCD [47], finding a mean score of 18.6 ± 7.6 in patients with novo/exacerbated OCD induced by clozapine.
- **OCI**: One study [52] used this scale in the measurement of OCD, reporting a mean score of 41 ± 30.6, as well as reporting the specific scores of each subscale in patients with schizophrenia receiving clozapine treatment and comparing them with a group of patients diagnosed with OCD.
- **OCI-R**: Three studies employed the OCI-R as an instrument for measuring OCD, finding a similar mean score among the studies: 20.3 ± 13.7 [28]; 19 ± 13.4 [54], and 18.4 ± 13.6 [56].
- **NIMH-GOCS**: One study used the NIMH-GOCS [48] as a measurement instrument, in addition to including the Y-BOCS; in this, a mean score of 6 ± 3.2 was obtained.

### Characteristics of obsessive-compulsive symptoms

A total of 7 studies describes the characteristics of OCS in their participants (Table 5). In Gálvez-Buccollini et al. [31], OCS was described using the Y-BOCS items. In the group treated with clozapine (56 participants), the most frequent obsessions were those of aggression (21.4%), various (17.9%), and contamination (16.1%). When comparing them with the group receiving treatment with classical antipsychotics (54 participants), it was found that aggression obsessions were the only ones that showed a statistically significant difference between both groups. Regarding compulsions, the most common in both groups were cleaning and various.

**Table 5:** Characteristics of obsessive-compulsive symptoms

Author	Assessment Tool	Obsessive-Compulsive Symptom	% of participants	Mean Score
**Gálvez-** **Buccollini **et al.	Y-BOCS	**Obsessions** ● Aggression ● Miscellaneous ● Contamination ● Sexual ● Symmetry ● Somatic ● Religious **Compulsions** ● Miscellaneous ● Cleaning ● Checking ● Repetitive rituals ● Arranging ● Counting ● Hoarding	21,4% 17,9% 16,1% 14,3% 5,4% 3,6% 5,4% 17,9% 17,9% 12,5% 5,4% 2,4% 0% 2,4%	-
**Gürcan **et al.	Interviews + Y-BOCS	● Checking: locks, stove, appliances ● Repetitive rituals: Need to know or remember certain things ● Miscellaneous: Need to tell, ask or confess ● Checking: Checking that did not make a mistake ● Counting compulsions ● Washing: Excessive or ritualized handwashing	85% 39% 33% 28% 20% 22%	-
**Doyle **et al.	OCI	● Washing, ● Checking ● Doubting ● Ordering ● Obsessing ● Hoarding ● Mental neutralising	-	0,74 ± 0,89 1,12 ± 0,87 1,30 ± 1,14 0,86 ± 1,14 1,17 ± 0,92 0,93 ± 0,92 0,95 ± 0,89
**Fernandez-Egea** ***et al. (2018)***	OCI-R	● Checking ● Obsessing ● Hoarding ● Ordering ● Neutralising ● Washing	-	5,1 ± 3,6 4,8 ± 3,6 3,2 ± 2,8 2,9 ± 3,0 2,4 ± 3,0 1,8 ± 2,4
**Parkin **et al.	OCI-R	● Washing ● Obsessional thinking ● Hoarding ● Ordering ● Checking ● Mental neutralising	-	1,72 ± 2,38 4,35 ± 3,51 3,2 ± 2,85 2,62 ± 2,83 4,72 ± 3,57 2,35 ± 2,9
**Fernandez-Egea ** ***et al. (*** **2024)**	OCI-R	● Washing ● Obsessing ● Hoarding ● Ordering ● Checking ● Neutralising	13,8% 42,1% 27,2% 24,1% 43,6% 20,0%	1,73 ± 2,40 4,22 ± 3,49 3,06 ± 2,77 2,56 ± 2,77 4,49 ± 3,50 2,37 ± 3,06
**Ertugrul **et al.	MOCI	● Checking ● Washing ● Doubting ● Slowness	-	5,2 ± 2,3 3,8 ± 2,3 4,4 ± 1,7 3,3 ± 1,6

*The table only includes patients with schizophrenia undergoing clozapine treatment

Ertugrul et al. [47] evaluated the types of OCS according to the subscales of the MOCI; in this study, they found that the checking subscale score was significantly higher in the group whose OCS was initiated or exacerbated by clozapine and in the group with preexisting OCS compared to the group that, despite treatment with clozapine, did not develop significant OCS.

Doyle et al. [52] evaluated OCS using the OCI in patients diagnosed with schizophrenia treated with clozapine and patients diagnosed with OCD. Their findings report that 14 participants (23%) in the clozapine group presented total OCI scores in a clinically significant range, although none of the OCI subscales in this group reached a clinically significant range. Nevertheless, the doubt subscale got the highest score in the clozapine group.

Fernandez-Egea et al. [28] reported OCS according to the OCI-R scale; in this study, they differentiated the total score of patients treated solely with clozapine (monotherapy) and patients treated with clozapine plus other medications (polypharmacy). The six subscales’ scores showed greater severity in the polypharmacy group, with a total OCI-R score of 24.6 (*p* = 0.001).

Unlike the other studies exploring the characteristics of OCS in their samples, Gürcan et al. [53] conducted this analysis not only using one scale (Y-BOCS) but also through electronic or physical records and clinical interviews, finding that checking compulsions, such as checking locks, stoves, or other appliances, were more frequent in the group of patients with clozapine-induced OCS.

Parkin et al. [54] evaluated the subscales of the OCI-R and their relationship with the impact on the well-being of patients with OCS. They found that the scores obtained in the subscales of obsessions and hoarding were related to a detrimental impact on the well-being of these patients.

Finally, Fernandez-Egea et al. [56] detailed the results of the subscales of the OCI-R and their association with the presence of psychotic symptoms, finding that psychotic symptoms in patients with schizophrenia significantly correlated with the severity of OCD and with the subscales of obsessions and checking.

### Treatment characteristics

#### Average clozapine dose

The average dose of clozapine in each studied sample was reported in ten of the 14 studies, where the average dose was in a range from 196 to 525 mg/day. In two studies, the overall dose was not reported; rather, the doses for patients who developed OCS were noted, such as Lin et al. [48], where the average dose for patients who developed OCS was 305.1 ± 157.2 mg, and Seow et al. [55], where the average dose was 337.5 ± 165.3 mg, while for those who developed OCD it was 325 ± 148.5 mg (Table 3).

#### Clozapine treatment duration

The average treatment duration with clozapine in each studied sample was reported in 11 of the 14 studies, where the average duration was found to range from five to 210 months of treatment (Table 3). On the other hand, Seow et al. [55], did not report for the general sample; rather, only for patients who developed OCS (54.8 ± 60.8 months) and for patients who developed OCD (59 ± 54.6 months). Finally, in the remaining two studies, the treatment duration was not reported. It should be noted that, in some cases, the duration was converted by researchers from years or days to months to standardize the information, which is marked with an (*) in Table 3.

#### Plasma clozapine concentration

The plasma concentration of clozapine was described in three of the 14 studies. In Lin et al. [48], reported the plasma concentration of clozapine and its metabolites (norclozapine and clozapine-N-oxide), finding that in patients who developed OCS, the concentration was higher than in those who did not: clozapine: 595.1 vs. 433.5 ng/mL, norclozapine: 266.4 vs. 203.1 ng/mL, clozapine-N-oxide: 59.6 vs. 54.2 ng/mL.

Gürcan et al. [53] reported the clozapine and norclozapine concentrations for the general sample. It was found that the concentration of clozapine was 828.11 ± 445.5 ng/mL, while that of norclozapine was 364.66 ± 200.17 ng/mL. Additionally, this study divided the sample into three groups: group A (patients who developed de novo OCS after treatment with clozapine) had a concentration of 815.6 ± 561.86 ng/mL for clozapine and 362.21 ± 198.77 ng/mL for norclozapine, while group B (patients who maintained their OCS without changes before and after using clozapine) had a concentration of 856.54 ± 432.93 ng/mL for clozapine and 363.55 ± 202.75 ng/mL for norclozapine; finally, group C (patients who did not develop OCS) had a concentration of 777.46 ± 425.25 ng/mL for clozapine and 363.31 ± 206.64 ng/mL for norclozapine. Differences between the concentrations of the three groups were not statistically significant.

Seow et al. [55] reported that the concentration of clozapine for patients who developed OCS was 915.2 ± 522.8 ng/mL, while the concentration for those who did not develop OCS was 844.8 ± 520.3 ng/mL. On the other hand, the concentration for those who developed OCD was 880.6 ± 459.1 ng/mL, and for those who did not, it was 857 ± 529.7 ng/mL.

### Clinical impact of obsessive-compulsive symptoms

The studies assessed the clinical impact of obsessive-compulsive symptoms (OCS) in patients with schizophrenia treated with clozapine. Parkin et al. [54] evaluated participants’ mental well-being using the Short Warwick–Edinburgh Mental Wellbeing Scale (SWEMWBS) and found that the presence of OCS was significantly associated with lower mental well-being. This negative association persisted even after controlling for depressive and psychotic symptoms. When evaluating the different types of OCS, it was found that obsessive thoughts and hoarding behaviors had a particularly negative impact on participants’ mental well-being.

On the other hand, Fernandez-Egea et al. [56] employed the Positive and Negative Syndrome Scale (PANSS) to assess the severity of psychotic symptoms in patients with schizophrenia treated with clozapine. They found that psychotic symptoms were significantly correlated with the severity of OCS, particularly with the obsession and checking compulsion subscales of the Obsessive-Compulsive Inventory-Revised (OCI-R).

### Methodological quality assessment

The methodological quality of the studies was assessed using the JBI’s Critical Appraisal Tools [43]. The evaluated aspects included sample inclusion criteria, reliability of exposure measurement, and the presence of confounding factors, among others. These are detailed in Table 6.

**Table 6:** Methodological quality assessment

#	Author	Were the criteria for inclusion in the sample clearly defined?	Were the study subjects and the setting described in detail?	Was the exposure measured in a valid and reliable way?	Were objective, standard criteria used for measurement of the condition?	Were confounding factors identified?	Were strategies to deal with confounding factors stated?	Were the outcomes measured in a valid and reliable way?	Was appropriate statistical analysis used?	Overall appraisal
1	Ghaemi et al. 1995	Yes	Yes	Yes	Yes	Yes	No	Yes	Yes	Include
2	Gálvez-Buccollini et al. 2004	Yes	Yes	Yes	Yes	No	Not applicable	Yes	Yes	Include
3	Ertugrul et al. 2005	Yes	Yes	Yes	Yes	No	Not applicable	Yes	Yes	Include
4	Lin et al. 2006	Yes	Yes	Yes	Yes	Yes	Yes	Yes	Yes	Include
5	Sa et al. 2009	Yes	Yes	Yes	Yes	Yes	Yes	Yes	Yes	Include
6	Bleakley et al. 2011	Yes	Yes	Yes	Yes	No	Not applicable	Yes	Yes	Include
7	Grassi et al. 2013	Yes	Yes	Yes	Yes	Yes	Yes	Yes	Yes	Include
8	Doyle et al. 2014	Yes	Yes	Yes	Yes	Yes	No	Yes	Yes	Include
9	Fernandez-Egea et al. 2018	Yes	Yes	Yes	Yes	Yes	Yes	Yes	Yes	Include
10	Gürcan et al. 2021	Yes	Yes	Yes	Yes	Yes	Yes	Yes	Yes	Include
11	Parkin et al. 2022	Yes	Yes	Yes	Yes	Yes	Yes	Yes	Yes	Include
12	Seow et al. 2023	Yes	Yes	Yes	Yes	No	Not applicable	Yes	Yes	Include
13	Fernandez-Egea et al. 2024	No	Yes	Yes	Yes	Yes	Yes	Yes	Yes	Include
14	Morgenroth C. et al. 2024	Yes	Yes	Yes	Yes	Yes	No	Yes	Yes	Include

## Discusion

### Prevalence of obsessive-compulsive symptoms

The overall prevalence of OCS showed wide variability, ranging from 20 to 76%, with a median of 38.55% and an interquartile range of 21.8% across eight of the 14 studies [47–49, 52, 54–57]. Comparatively, other reviews also reported wide variability in prevalence: Grillaut et al., who evaluated a broad range of antipsychotics (clozapine, olanzapine, risperidone, quetiapine, aripiprazole, and ziprasidone), found that among 18 studies focused exclusively on clozapine-treated patients, OCS prevalence ranged from 6.9 to 46.4%, including case reports and case series [26]. Similarly, the review by Fonseka et al., which included 15 studies exclusively with clozapine-treated patients, reported a prevalence of 20–28% for the development of de novo OCS and 10–18% for the exacerbation of pre-existing OCS, without reaching a clear conclusion on the overall development of OCS [27].

The variability in prevalence observed in the studies included in this review may be attributed to several factors. First, sample size is a key consideration; the eight studies evaluated OCS in fewer than 80 schizophrenia patients treated with clozapine, which may limit the precision of their estimates. Secondly, the different scales used across studies to assess the presence of OCS could have contributed to this variability. Additionally, the demographic and clinical characteristics of the studied populations may have influenced the differences observed. Finally, variation in the average dose and duration of clozapine treatment across studies could also have affected the results. These factors will be discussed in more detail in the following sections. It is important to consider that, in many cases, patients who develop OCS in the context of clozapine use are treated with medications aimed at reducing these symptoms, such as SSRIs, TCAs, etc., with the goal of achieving clinical improvement. However, studies by Fernández-Egea et al., Lin et al., and Gürcan et al. [28, 48, 53] found that significantly higher prevalence rates and scale scores were observed in patients using medications intended to reduce OCS. It is important to note that these studies did not report the dosage or duration of treatment with these medications, which precludes a detailed analysis of their influence.

In addition to clinical and demographic factors, genetic factors may also play a crucial role in the predisposition to developing OCS in these patients. The study by Morgenroth et al. [57], included in this review, investigated polygenic risk scores (PRS) in this population and found a significant correlation between PRS related to clozapine metabolism and the emergence of OCS. PRS are weighted sums of genetic variants used to estimate an individual’s risk of developing certain disorders or diseases. In the context of this study, PRS may help predict the genetic predisposition of patients to develop OCS/OCD. The study highlights that variations in genes related to clozapine metabolism, particularly in the CYP1A2 enzyme, could alter plasma levels of the drug and thereby influence the development of OCS/OCD. This genetic perspective underscores the importance of moving towards a more personalized approach in the treatment of schizophrenia, which considers both genetic predisposition and metabolic response to antipsychotics.

### Description and evaluation of obsessive-compulsive symptoms

#### Types of obsessive-compulsive symptoms

The studies included in this review reported a variety of obsessive-compulsive symptoms (OCS) in patients with schizophrenia treated with clozapine. The most reported symptoms were aggressive, miscellaneous, and contamination obsessions [31], as well as checking compulsions [47, 53]. For instance, Gálvez-Buccollini et al. [31] found that aggressive, miscellaneous, and contamination obsessions were significantly more prevalent in clozapine-treated patients compared to those treated with typical antipsychotics.

On the other hand, Fernandez-Egea et al. [56] explored the hypothesis that schizophrenia patients treated with clozapine tend to develop checking compulsions. To investigate this hypothesis, they conducted an exploratory analysis of the relationship between psychosis and excessive checking behavior. They found that, during the remission phase of psychosis, the severity of checking compulsions (measured using the OCI-R scale) correlated with both the dose and plasma concentration of clozapine.

These findings highlight the importance of conducting longitudinal studies that evaluate the progression of OCS over time and during different treatment phases to gain a clearer understanding of their development and management in schizophrenia patients treated with clozapine. This could help identify more effective and personalized intervention strategies.

#### Severity of obsessive-compulsive symptoms

The literature reports various scales for the evaluation of obsessive-compulsive symptoms (OCS) in the general population, with the most widely used being the Yale-Brown Obsessive-Compulsive Scale (Y-BOCS) and the Obsessive-Compulsive Inventory-Revised (OCI-R). Among these, the Y-BOCS is the most frequently employed scale for measuring the severity of OCS [37].

In this review, the severity of OCS was primarily assessed across studies using the Y-BOCS, with reported scores ranging from 10 ± 4.7 to 21.5 ± 6.69. These scores suggest that OCS may manifest clinically significantly, from mild to moderate, in patients with schizophrenia treated with clozapine [58]. In studies using the OCI-R scale, the mean scores and corresponding standard deviations were similar, around 18 to 20 points, indicating moderate severity according to the established cut-off points for OCS [59].

#### Comparison between monotherapy and polypharmacy

It is important to mention that patients diagnosed with schizophrenia may receive more than one psychotropic medication based on the individual’s symptoms and evaluation. Antipsychotics are the drugs of choice for treating the psychotic symptoms of schizophrenia, and they are often combined with other medications. For example, an antidepressant may be added to address negative symptoms, a benzodiazepine for comorbid anxiety, or a mood stabilizer in cases of significant mood instability [60]. In clinical practice, the use of two or more antipsychotics is common, with estimates suggesting that 10–20% of outpatient schizophrenia patients and up to 40% of hospitalized patients receive polypharmacy, although these percentages may vary depending on the population studied [61].

This practice is evident when analyzing the studies included in this review. Fernandez-Egea et al. [28] considered the use of concomitant medications alongside clozapine in their analysis, finding higher scores on the OCI-R subscales compared to those treated with clozapine alone. This finding suggests that the addition of other medications may enhance the appearance or severity of obsessive-compulsive symptoms (OCS), underscoring the importance of considering polypharmacy as a potential risk factor in the evaluation and management of OCS in patients with schizophrenia. However, it is worth noting that this could be a confounding factor, as patients requiring more concomitant medications—such as aripiprazole, selective serotonin reuptake inhibitors (SSRIs), serotonin-norepinephrine reuptake inhibitors (SNRIs), or tricyclic antidepressants (TCAs)—may present with more severe baseline OCS. This would imply that the addition of these medications is an attempt to control these symptoms rather than polypharmacy itself causing an increase in OCS.

#### Clinical impact of obsessive-compulsive symptoms

Two studies evaluated the clinical impact of obsessive-compulsive symptoms (OCS) in patients with schizophrenia treated with clozapine. Parkin et al. [54] found that the presence of OCS was significantly associated with lower mental well-being, and this negative association persisted even after controlling for depressive and psychotic symptoms. On the other hand, Fernandez-Egea et al. [56] found that psychotic symptoms were significantly correlated with the severity of OCS and with the obsession and checking compulsion subscales of the OCI-R. These findings are consistent with prior literature, as demonstrated by a meta-analysis conducted in 2009, which assessed the association between the severity of OCS and psychotic symptoms, finding that the presence of OCS was associated with greater severity of overall, positive, and negative psychotic symptoms [62].

### Treatment characteristics

#### Dose, duration and adherence to treatment with clozapine

**Fig. 5:**
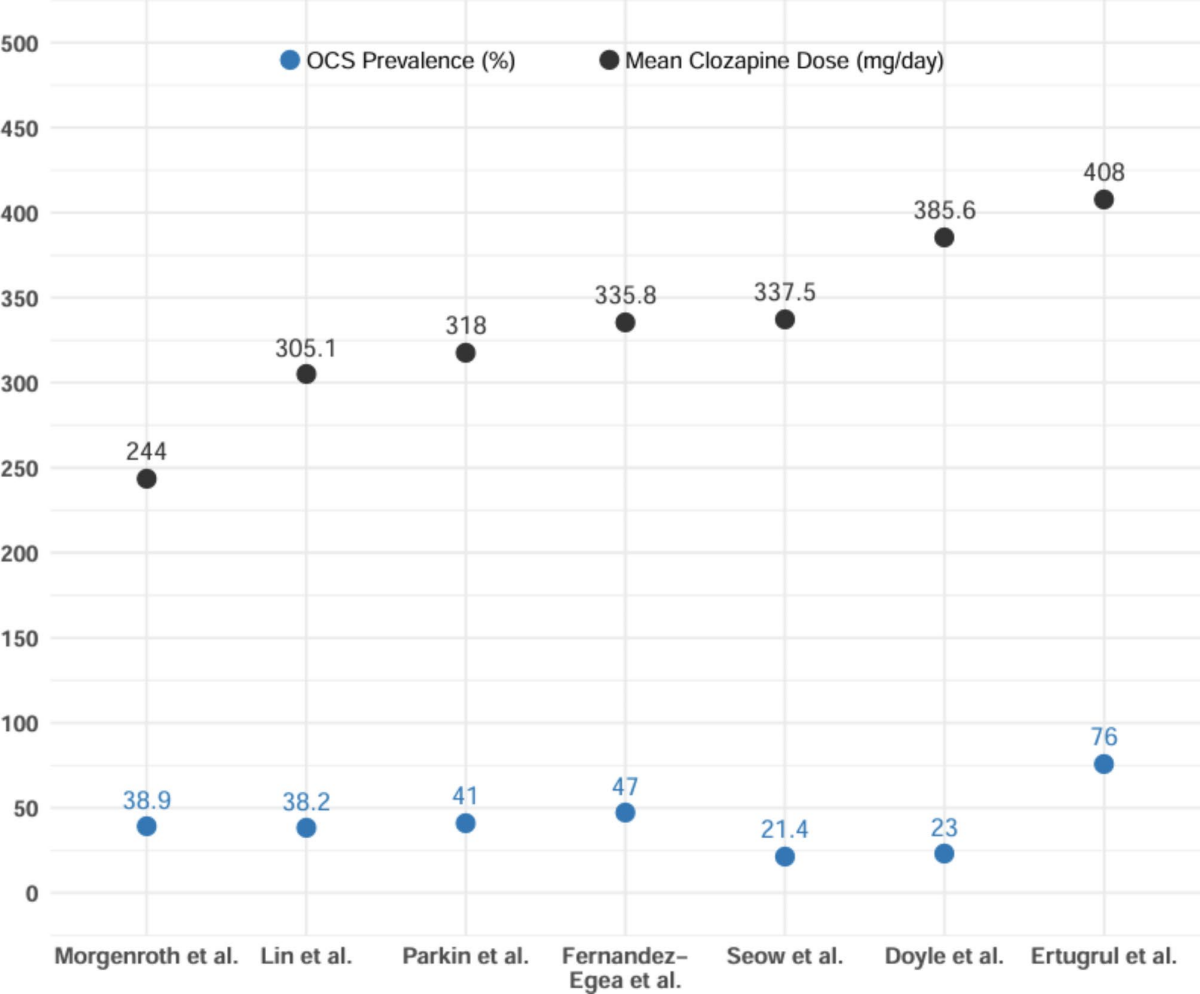
Comparison of OCS prevalence and mean dose of clozapine

Studies examining the relationship between the dose and duration of clozapine treatment and the development of obsessive-compulsive symptoms (OCS) show mixed results. In Ghaemi et al. [46], no significant relationship was found between the dose and duration of treatment and the development of OCS/OCD; however, when compared to the study by Baker et al. [63], it was noted that the clozapine dose used in Ghaemi et al. study (291.2 ± 183.9 mg/day vs. 630 ± 145 mg/day) was considerably lower, and the treatment duration was also shorter (5 ± 7.8 months vs. 7 ± 5.2 months). The difference in dosage was statistically significant, suggesting that higher doses of clozapine might be associated with an increased risk of developing OCS, while the treatment duration did not show a significant difference in relation to OCS (Fig. 5).

On the other hand, Gálvez-Bucollini et al. [31] provides a different perspective. When comparing the effects of clozapine treatment versus conventional antipsychotics on the occurrence of OCS, they found that the treatment duration was longer in patients who developed OCS compared to those who did not (50 ± 55.2 months vs. 28.5 ± 39 months). However, no significant difference was found regarding the dose of clozapine. This suggests that, in this case, the duration of exposure to clozapine may play a more relevant role than the dosage in the onset of OCS.

Given the discrepancies observed across studies, Kim et al. [64] proposed a hypothesis suggesting that the relationship between clozapine dosage and the emergence of obsessive-compulsive symptoms (OCS) may follow a U-shaped curve rather than a linear pattern. This nonlinear association is posited to be mediated by dose-dependent alterations in 5HT2A and D2 receptor occupancy. According to this hypothesis, both low and high doses of clozapine could be associated with the emergence of OCS, but through different mechanisms. At low doses, clozapine may saturate 5-HT2A receptors more than D2 receptors, increasing the occupancy ratio of 5-HT2A/D2 receptors, which could contribute to the emergence of OCS. At mid doses (around 300 mg/d), although D2 receptor occupancy increases, the 5-HT2A/D2 ratio decreases, which might reduce the emergence of OCS. Conversely, at doses higher than 300 mg/day, the relationship becomes positive again. As previously discussed, with increasing clozapine doses, D2 receptor occupancy rises relative to 5-HT2A receptor occupancy, leading to a decrease in the 5-HT2A/D2 ratio. Unlike the 5-HT2A receptors, clozapine does not fully saturate D2 receptors (65–68%), and at doses above approximately 300 mg/day, the 5-HT2A/D2 ratio remains relatively stable. Additionally, it is worth noting that dosage is not an appropriate way to measure exposure to clozapine, as it can be influenced by treatment adherence and various factors that affect its pharmacokinetics [28]. In summary, the results regarding clozapine dosage and the emergence of OCS remain unclear, suggesting the need for more detailed studies using different methodologies to verify these theories. Regarding adherence to treatment, none of the studies explicitly evaluated this aspect; however, some implied it in their inclusion criteria or methods. Sa et al. [49] indicated that patients had to be “stable” on their dose of clozapine or haloperidol for six months prior to the interview. Galvez-Bucollini et al. [31] specified that patients must have been receiving their antipsychotic treatment “regularly” at the time of evaluation.

#### Clozapine plasma concentration

In the study conducted by Lin et al. [48], the plasma concentrations of clozapine and its metabolites (norclozapine and clozapine-N-oxide) were evaluated, revealing that patients who developed obsessive-compulsive symptoms (OCS) exhibited higher concentrations compared to those who did not (clozapine: 595.1 vs. 433.5 ng/mL, norclozapine: 266.4 vs. 203.1 ng/mL, clozapine-N-oxide: 59.6 vs. 54.2 ng/mL). A quartile-based odds ratio comparison demonstrated that, despite no significant differences in the administered dose between the groups, individuals with higher plasma concentrations of clozapine and norclozapine had a fourfold increased risk of developing OCS. Conversely, clozapine-N-oxide was not associated with a significant risk. The study further observed that the plasma concentrations of clozapine and norclozapine were elevated in patients receiving concomitant treatment with selective serotonin reuptake inhibitors (SSRIs) or mood stabilizers, possibly due to pharmacokinetic interactions, although further studies are recommended to validate this hypothesis.

These findings contrast with those of Seow et al. [55], who did not identify a significant association between clozapine plasma concentrations and the development of OCS or obsessive-compulsive disorder (OCD) in schizophrenia patients treated with clozapine. Such discrepancies may be attributed to differences in study design, methodological approaches, sample size, or variability in patient characteristics. These divergent results underscore the necessity for further research to elucidate the relationship between clozapine plasma concentrations and the emergence of OCS in this population.

It is essential to acknowledge that several factors, such as body mass index (BMI), smoking status, and concomitant medications, can influence clozapine plasma levels [65]. Lin et al. [48] compared plasma concentrations between smokers and non-smokers, finding that smoking significantly reduced clozapine and norclozapine levels (clozapine from 549.3 ± 328.6 to 387.2 ± 234.9 ng/mL; norclozapine from 235.9 ± 135.8 to 210 ± 126.4 ng/mL). These findings are consistent with previous research demonstrating the impact of smoking on clozapine pharmacokinetics [65, 66].

Studies investigating the relationship between clozapine plasma concentrations and the development of OCS/OCD should carefully consider intrinsic patient factors, such as smoking habits, which can significantly alter clozapine and its metabolite levels. The variability in findings across studies highlights the need to control for these confounding factors in future research to draw more robust and clinically applicable conclusions.

### Strengths and limitations

An important strength of this review lies in the use of multiple databases to identify the studies for inclusion, as well as the incorporation of research from various countries, which enhances the geographic diversity of the analyzed sample. Additionally, this review did not limit itself to reporting only the prevalence of obsessive-compulsive symptoms (OCS) in patients with schizophrenia treated with clozapine; it also explored treatment characteristics, types and severity of symptoms, and their relationship with dosage and plasma concentration.

However, this review has several important limitations. First, the heterogeneity of the included studies complicates direct comparisons and limits the possibility of synthesizing the results through meta-analysis. Furthermore, the lack of prospective studies represented a significant constraint, limiting the ability to establish robust causal relationships. Secondly, some studies included participants who were receiving medications in addition to clozapine, which may have confounded the specific association between clozapine and OCS.Another relevant limitation is the incomplete understanding of clozapine’s dose dependent mechanisms of action that could influence the interpretations of the reported prevalence of OCS in patients treated with this medication. Medication adherence was not explicitly evaluated in the studies, making it difficult to ascertain the extent to which non-adherence may have influenced the reported outcomes. While some studies included criteria implying regular treatment, the lack of a direct assessment introduces potential variability and limits the robustness of the findings.

Another important limitation of this scoping review is the lack of evaluation of psychotherapy or counseling interventions as potential protective factors against obsessive-compulsive symptoms as these non-pharmacological interventions were not explicitly explored in the studies included. These interventions, often employed to manage or alleviate symptoms, might influence the manifestation or reporting of OCD symptoms and could potentially affect the prevalence estimates of this review. Future research should consider investigating the impact of psychotherapy and counseling in this context to provide a more comprehensive understanding.

Finally, the small sample size in certain studies reduced the statistical power of the analyses and restricted the generalizability of the findings.

### Recommendations

The findings reported in this study underscore the importance of monitoring obsessive-compulsive symptoms (OCS), whether newly arising or exacerbated, in patients with schizophrenia undergoing treatment with clozapine. Given the wide range of reported prevalence rates, it is crucial for treating physicians to consider the possibility that OCS may emerge or intensify during clozapine treatment and to develop individualized management strategies for these patients.

For future research, it is recommended that longitudinal prospective studies be conducted to assess the evolution of OCS in this patient population, employing standardized tools for measuring OCS and controlling for other factors that may influence this relationship, such as the concomitant use of other medications (antipsychotics, antidepressants, etc.), dosage, duration of treatment, and plasma concentration of clozapine, as well as individual factors such as genetics and smoking habits. The relationship between clozapine dosage and treatment duration and the development of OCS is complex and not yet fully understood. While some studies suggest that higher doses of clozapine may be associated with an increased risk of OCS, others indicate that the duration of treatment may be a more relevant factor. The U-shaped hypothesis suggests that both low and high doses of clozapine can trigger OCS through different mechanisms. At low doses, greater occupancy of 5-HT2A receptors compared to D2 receptors may increase the 5-HT2A/D2 ratio, potentially contributing to the development of OCS. In contrast, at mid-range doses (around 300 mg/day), increased D2 receptor occupancy may reduce the 5-HT2A/D2 ratio, potentially alleviating OCS, although other factors may still play a role. At higher doses, the relationship shifts again, with increased D2 receptor occupancy compared to 5-HT2A receptors, leading to a decrease in the 5-HT2A/D2 ratio. This hypothesis highlights the need for further research to clarify these mechanisms Such research will contribute to a better understanding of the relationship between clozapine and OCS and ultimately help optimize management and improve the quality of life for patients.

## Conclusions

The prevalence of obsessive-compulsive symptoms (OCS) in patients with schizophrenia treated with clozapine exhibits significant variability, ranging from 20 to 76%, with a median of approximately 37.9%. This variability can be attributed to differences in sample size, assessment methodologies, and the demographic and clinical characteristics of the studied populations, as well as variations in doses and duration of clozapine treatment. Despite these differences, studies suggest that clozapine, as an antagonist of the 5-HT2A receptors, may contribute to the development of OCS due to its impact on serotonergic regulation. However, it is important to emphasize that the mechanism of action is not completely understood, as it can be influenced by various factors.

The most frequently reported types of OCS include obsessions related to aggression and checking. Assessment scales, such as the Yale-Brown Obsessive-Compulsive Scale (Y-BOCS) and the Obsessive-Compulsive Inventory-Revised (OCI-R), indicate that the severity of OCS in this population can range from mild to moderate. Patients with schizophrenia treated with clozapine who are also on polypharmacy tend to score higher on OCS scales, suggesting that the concomitant use of other medications may influence the severity of OCS. The clinical impact of OCS is significant, adversely affecting mental well-being and correlating with increased severity of psychotic symptoms.

Furthermore, the plasma concentration of clozapine appears to play an important role, with studies suggesting that higher concentrations are associated with an increased risk of OCS, although results are not uniform and may be influenced by individual patient factors.

## Electronic Supplementary Material

Below is the link to the electronic supplementary material.

Supplementary Material 1

## Data Availability

The data supporting this scoping review are derived from the 14 studies analyzed and are listed in Table 2. Further details can be obtained from the corresponding author upon reasonable request.

## Abbreviations

OCS: Obsessive-compulsive symptoms
OCD: Obsessive-compulsive disorder
TRS: Treatment-resistant schizophrenia
SSRIs: Selective serotonin reuptake inhibitors
SRIs: Serotonin reuptake inhibitors
SNRIs: Serotonin-norepinephrine reuptake inhibitors
TCAs: Tricyclic antidepressants
PRISMA-ScR: Preferred Reporting Items for Systematic Reviews and Meta-Analyses extension for Scoping Reviews
MeSH: Medical Subject Headings
DeCS: Health Sciences Descriptors
Y-BOCS: Yale-Brown Obsessive-Compulsive Scale
MOCI: Maudsley Obsessive-Compulsive Inventory
OCI: Obsessive-Compulsive Inventory
OCI-R: Obsessive-Compulsive Inventory - Revised
NIMH-GOCS: National Mental Health Institute Global Obsessive-Compulsive Scale
IQR: Interquartile range
SWEMWBS: Short Warwick–Edinburgh Mental Wellbeing Scale
PANSS: Positive and Negative Syndrome Scale
