# Supplementary material for: Prevalence of obsessive-compulsive symptoms in patients with schizophrenia treated with clozapine: a scoping review
Source: BMC Psychiatry. 2025 Jan 23;25:71. doi: 10.1186/s12888-024-06466-9 (PMC11759428; doi:10.1186/s12888-024-06466-9)
Supplement: Supplementary file 1 — Supplementary Material 1 [file 12888_2024_6466_MOESM1_ESM.docx]

**Appendix**

**Appendix 1:** PRISMA-ScR’s verification list

| SECTION | ITEM | PRISMA-ScR CHECKLIST ITEM | REPORTED ON PAGE # |
| --- | --- | --- | --- |
| TITLE | | | |
| Title | 1 | Identify the report as a scoping review. | 1 |
| ABSTRACT | | | |
| Structured summary | 2 | Provide a structured summary that includes (as applicable): background, objectives, eligibility criteria, sources of evidence, charting methods, results, and conclusions that relate to the review questions and objectives. | 12 |
| INTRODUCTION | | | |
| Rationale | 3 | Describe the rationale for the review in the context of what is already known. Explain why the review questions/objectives lend themselves to a scoping review approach. | 16 |
| Objectives | 4 | Provide an explicit statement of the questions and objectives being addressed with reference to their key elements (e.g., population or participants, concepts, and context) or other relevant key elements used to conceptualize the review questions and/or objectives. | 24 |
| METHODS | | | |
| Protocol and registration | 5 | Indicate whether a review protocol exists; state if and where it can be accessed (e.g., a Web address); and if available, provide registration information, including the registration number. | 25 |
| Eligibility criteria | 6 | Specify characteristics of the sources of evidence used as eligibility criteria (e.g., years considered, language, and publication status), and provide a rationale. | 25 |
| Information sources | 7 | Describe all information sources in the search (e.g., databases with dates of coverage and contact with authors to identify additional sources), as well as the date the most recent search was executed. | 26 |
| Search | 8 | Present the full electronic search strategy for at least 1 database, including any limits used, such that it could be repeated. | 92 |
| Selection of sources of evidence | 9 | State the process for selecting sources of evidence (i.e., screening and eligibility) included in the scoping review. | 26 |
| Data charting process | 10 | Describe the methods of charting data from the included sources of evidence (e.g., calibrated forms or forms that have been tested by the team before their use, and whether data charting was done independently or in duplicate) and any processes for obtaining and confirming data from investigators. | 28 |
| Data items | 11 | List and define all variables for which data were sought and any assumptions and simplifications made. | 28 |
| Critical appraisal of individual sources of evidence | 12 | If done, provide a rationale for conducting a critical appraisal of included sources of evidence; describe the methods used and how this information was used in any data synthesis (if appropriate). | 31 |
| Synthesis of results | 13 | Describe the methods of handling and summarizing the data that were charted. | 31 |
| RESULTS | | | |
| Selection of sources of evidence | 14 | Give numbers of sources of evidence screened, assessed for eligibility, and included in the review, with reasons for exclusions at each stage, ideally using a flow diagram. | 32 |
| Characteristics of sources of evidence | 15 | For each source of evidence, present characteristics for which data were charted and provide the citations. | 75 |
| Critical appraisal within sources of evidence | 16 | If done, present data on critical appraisal of included sources of evidence (see item 12). | 73 |
| Results of individual sources of evidence | 17 | For each included source of evidence, present the relevant data that were charted that relate to the review questions and objectives. | 78 |
| Synthesis of results | 18 | Summarize and/or present the charting results as they relate to the review questions and objectives. | 32 |
| DISCUSSION | | | |
| Summary of evidence | 19 | Summarize the main results (including an overview of concepts, themes, and types of evidence available), link to the review questions and objectives, and consider the relevance to key groups. | 46 |
| Limitations | 20 | Discuss the limitations of the scoping review process. | 55 |
| Conclusions | 21 | Provide a general interpretation of the results with respect to the review questions and objectives, as well as potential implications and/or next steps. | 58 |
| FUNDING | | | |
| Funding | 22 | Describe sources of funding for the included sources of evidence, as well as sources of funding for the scoping review. Describe the role of the funders of the scoping review. | 7 |

**Appendix 2:** Research strategy

PubMed

| “Obsessive-Compulsive Disorder” [MeSH] | - Disorder, Obsessive-Compulsive - Disorders, Obsessive-Compulsive - Obsessive Compulsive Disorder - Obsessive-Compulsive Disorders - Anankastic Personality - Anankastic Personalities - Personalities, Anankastic - Personality, Anankastic - Neurosis, Obsessive-Compulsive - Neuroses, Obsessive-Compulsive - Neurosis, Obsessive Compulsive - Obsessive-Compulsive Neuroses - Obsessive-Compulsive Neurosis |
| --- | --- |
| “Obsessive Behavior” [MeSH] | - Behavior, Obsessive - Behaviors, Obsessive - Obsessive Behaviors - Obsessions - Obsession |
| “Compulsive Behavior” [MeSH] | - Behavior, Compulsive - Behaviors, Compulsive - Compulsive Behaviors |
| “Schizophrenia” [MeSH] | - Schizophrenias - Dementia Praecox - Schizophrenic Disorders - Disorder, Schizophrenic - Disorders, Schizophrenic - Schizophrenic Disorder |
| “Clozapine” [MeSH] | - Leponex - Clozaril |
| Free terms | - Obsessive compulsive disorder - Obsession - Compulsion - Obsessive compulsive symptom - Obsessive compulsive symptoms - Obsessive - Compulsive - Psychosis - Psychosis disorder - Schizophrenias - Schizophrenia diagnosis - Antipsychotic - Antipsychotic medication - Clozapine |
| MeSH + términos libres: ((((((((((((obsessive compulsive disorder) OR (obsession)) OR (compulsion)) OR (obsessive compulsive symptom)) OR (obsessive compulsive symptoms)) OR (obsessive)) OR (compulsive)) OR (Obsessive-Compulsive Disorder[MeSH Terms])) OR (Obsessive Behavior[MeSH Terms])) OR (Compulsive Behavior[MeSH Terms]))) AND ((((((schizophrenia) OR (psychosis)) OR (psychosis disorder)) OR (schizophrenias)) OR (schizophrenia diagnosis)) OR (Schizophrenia[MeSH Terms]))) AND ((((clozapine) OR (antipsychotic)) OR (antipsychotic medication)) OR (clozapine[MeSH Terms])) | |
| Search results: 613 estudios | |

Lilacs

| Schizophrenia (DeCS) | - Schizophrenia - Schizophrenia, Catatonic - Schizophrenia, Disorganized - Schizophrenia, Paranoid - Schizophrenia Spectrum and Other Psychotic Disorders |
| --- | --- |
| Obsessive-Compulsive Disorder (DeCS) | - Obsessive-Compulsive Disorder |
| Clozapine (DeCS) | - Clozapine |
| Free terms | - Disorder, Obsessive-Compulsive - Disorders, Obsessive-Compulsive - Obsessive Compulsive Disorder - Obsessive-Compulsive Disorders - Anankastic Personality - Anankastic Personalities - Personalities, Anankastic - Personality, Anankastic - Neurosis, Obsessive-Compulsive - Neuroses, Obsessive-Compulsive - Neurosis, Obsessive Compulsive - Obsessive-Compulsive Neuroses - Obsessive-Compulsive Neurosis - Obsessive Behavior - Behavior, Obsessive - Behaviors, Obsessive - Obsessive Behaviors - Obsessions - Obsession - Obsessive Compulsive Symptoms |
| DeCS + términos libres: ((Schizophrenia) OR (Schizophrenia, Catatonic) OR (Schizophrenia, Disorganized) OR (Schizophrenia, Paranoid) OR (Schizophrenia Spectrum and Other Psychotic Disorders)) AND ((Obsessive-Compulsive Disorder) OR (Disorder, Obsessive-Compulsive) OR (Disorders, Obsessive-Compulsive) OR (Obsessive Compulsive Disorder) OR (Obsessive-Compulsive Disorders) OR (Anankastic Personality) OR (Anankastic Personalities) OR (Personalities, Anankastic) OR (Personality, Anankastic) OR (Neurosis, Obsessive-Compulsive) OR (Neuroses, Obsessive-Compulsive) OR (Neurosis, Obsessive Compulsive) OR (Obsessive-Compulsive Neuroses) OR (Obsessive-Compulsive Neurosis) OR (Obsessive Behavior) OR (Behavior, Obsessive) OR (Behaviors, Obsessive) OR (Obsessive Behaviors) OR (Obsessions) OR (Obsession) OR (Obsessive Compulsive Symptoms)) AND (Clozapine) | |
| Search result: 160 | |

Scielo

| Esquizofrenia (DeCS) | - Esquizofrenia - Esquizofrenia Catatónica - Esquizofrenia Hebefrénica - Esquizofrenia Paranoide - Espectro de Esquizofrenia y Otros Trastornos Psicóticos |
| --- | --- |
| Trastorno Obsesivo Compulsivo (DeCS) | - Trastorno Obsesivo Compulsivo |
| Clozapina (DeCS) | - Clozapina |
| Free terms | - Trastorno Obsesivo-Compulsivo - Trastorno, Obsesivo-Compulsivo - Trastornos Obsesivo-Compulsivos - Personalidad Anancástica - Personalidades Anancásticas - Personalidades, Anancástica - Personalidad, Anancástica - Neurosis Obsesivo-Compulsiva - Neurosis, Obsesivo-Compulsiva - Neurosis Obsesiva Compulsiva - Comportamiento Obsesivo - Comportamiento, Obsesivo - Comportamientos Obsesivos - Comportamientos, Obsesivos - Obsesiones - Obsesión - Síntomas Obsesivo-Compulsivos |
| DeCS + términos libres: ((Esquizofrenia) OR (Esquizofrenia Catatónica) OR (Esquizofrenia Hebefrénica) OR (Esquizofrenia Paranoide) OR (Espectro de Esquizofrenia y Otros Trastornos Psicóticos)) AND ((Trastorno Obsesivo Compulsivo) OR ((Trastorno Obsesivo-Compulsivo) OR (Trastorno, Obsesivo-Compulsivo) OR (Trastornos Obsesivo-Compulsivos) OR (Personalidad Anancástica) OR (Personalidades Anancásticas) OR (Personalidades, Anancástica) OR (Personalidad, Anancástica) OR (Neurosis Obsesivo-Compulsiva) OR (Neurosis, Obsesivo-Compulsiva) OR (Neurosis Obsesiva Compulsiva) OR (Comportamiento Obsesivo) OR (Comportamiento, Obsesivo) OR (Comportamientos Obsesivos) OR (Comportamientos, Obsesivos) OR (Obsesiones) OR (Obsesión) OR (Yesntomas Obsesivo-Compulsivos)) AND (Clozapina) | |
| Search result: 0 | |

Embase

| # | Consulta | Resultados |
| --- | --- | --- |
| 1 | exp schizophrenia/ | 213,629 |
| 2 | exp obsessive compulsive disorder/ | 50,995 |
| 3 | clozapine/ | 38,412 |
| 4 | obsessive compulsive symptom*.mp. [mp=title, abstract, heading word, drug trade name, original title, device manufacturer, drug manufacturer, device trade name, keyword heading word, floating subheading word, candidate term word] | 3,482 |
| 5 | 2 or 4 | 51,379 |
| 6 | 1 and 3 and 5 | 697 |
| Search result: 697 | |  |
